# High Pressure Stress Response: Involvement of NMDA Receptor Subtypes and Molecular Markers

**DOI:** 10.3389/fphys.2019.01234

**Published:** 2019-09-27

**Authors:** Alice Bliznyuk, Michael Hollmann, Yoram Grossman

**Affiliations:** ^1^Zlotowski Center for Neuroscience, Department of Physiology and Cell Biology, Faculty of Health Sciences, Ben-Gurion University of the Negev, Beersheba, Israel; ^2^Israel Naval Medical Institute, Haifa, Israel; ^3^Department of Biochemistry I – Receptor Biochemistry, Faculty of Chemistry and Biochemistry, Ruhr University Bochum, Bochum, Germany

**Keywords:** HPNS, GluN2A, GAPDH, actin, high pressure biology, NMDAR, central nervous system, deep diving physiology

## Abstract

Professional divers who are exposed to high pressure (HP) above 1.1 MPa suffer from high pressure neurological syndrome (HPNS), which is characterized by reversible CNS hyperexcitability and cognitive and motor deficits. HPNS remains the final major constraints on deep diving at HP. Prolonged and repetitive exposure to HP during deep sea saturation dives may result in permanent memory and motor impairment. Previous studies revealed that CNS hyperexcitability associated with HPNS is largely induced by *N*-methyl-D-aspartate receptors (NMDARs). NMDARs that contain the GluN2A subunit are the only ones that show a large (∼60%) current increase at He HP. NMDAR subtypes that contain other GluN2 members show minor decrease or no change of the current. Immunoprecipitation was used in order to test the hypothesis that current augmentation may result from inserting additional NMDARs into the membrane during the 20–25 min compression. The results indicated that there is no increase in surface expression of NMDARs in the oocyte membrane under HP conditions. In contrast, consistent increase in glyceraldehyde-3-phosphate dehydrogenase (GAPDH) and β-actin was discovered. GAPDH and β-actin are cytosolic proteins which involve in various cellular control processes, increase of their expression suggests the presence of a general cellular stress response to HP. Understanding the precise hyperexcitation mechanism(s) of specific NMDAR subtypes and other possible neurotoxic processes during HP exposure could provide the key for eliminating the adverse, yet reversible, short-term effects of HPNS and hopefully the deleterious long-term ones.

## Introduction

Pressure, a fundamental state variable, tends to oppose any molecular interaction involving a positive change in volume, and vice versa. Hyperbaric pressure slows the rate of a reaction and increases the concentration of substances causing a given change. Pressure acts instantaneously throughout the organism and can affect any cell. The most common component of a eukaryotic organism is the bilayer membrane phospholipid. A pressure-dependent change in membrane phase-transition or volume/structure will impact a wide spectrum of biological processes (Daniels and Grossman, 2010). These changes lead in turn to secondary conformational alterations of the transmembranal voltage-dependent and ligand-gated ionic channels; of various metabolic receptors; of ionic and amino acid transporters; and of membrane-bound enzymes. Pressure may also act directly on all these components. At pressure, water-soluble proteins also display small negative volume change and their spatial coil structure undergoes transition and reversible denaturation. Interactions between large molecules that are associated with positive volume changes include self-assembly of polymeric proteins (such as muscular actin and myosin filaments) and protein–protein association. Many of the aforementioned proteins are crucial in controlling the excitability of neurons and their synaptic interactions within neuronal networks (for a review, see Grossman et al., 2010). A remarkable manifestation of high pressure (HP) physiological complexity is the high pressure neurological syndrome (HPNS) (Halsey, 1982; Bennett and Rostain, 2003; Grossman et al., 2010) observed among professional divers, as well as animals, during exposure to pressures above 1.1 MPa. HPNS is characterized by reversible central nervous system (CNS) hyperexcitability, cognitive deficits, autonomic nervous system (ANS) malfunction and motor problems (Brauer, 1984; Abraini, 1997). Professional divers performing deep-sea operations may also suffer from permanent memory and motor impairment (Gronning and Aarli, 2011), which could be associated with long-term HP toxicity. Previous studies revealed that one of the major contributors to CNS hyperexcitability in HPNS is *N*-methyl-D-aspartate receptor (NMDAR) hyperactivation (Fagni et al., 1985, 1987; Zinebi et al., 1988, 1990; Daniels and Grossman, 2003; Mor and Grossman, 2010). NMDAR is a member of the ionotropic glutamate receptor (iGluR) family, which also includes α-amino-3-hydroxy-5-methyl-4-isoxazolepropionic acid receptor (AMPAR) and kainate receptors. iGluRs convert transient glutamate release from presynaptic vesicles into postsynaptic neuronal excitation in synapses. This excitatory neurotransmission is one of the fundamental blocks needed for the correct development and function of the mammalian brain. Ca^2+^ influx through open NMDARs is pivotal to intracellular signaling, which is responsible for synaptic transmission plasticity during learning and memory (Traynelis et al., 2010). NMDAR-mediated Ca^2+^ influx is controlled by multiple patterns of heterotetramerization of GluN1 [eight subunits; GluN1-1a to -4a and GluN1-1b to -4b, resulting from alternative RNA splicing (Dingledine et al., 1999)]; GluN2 (four subunits, A-D, encoded by four different genes); and GluN3 (two subunits, A-B, encoded by two different genes). Hyper- and hypo-activation of NMDARs have been implicated in neurological disorders such as epilepsy, stroke, schizophrenia, and Alzheimer’s disease (Paoletti et al., 2013). Hyper-activation of NMDARs will allow excessive neuronal Ca^2+^ influx that may be associated with “glutamate toxicity” or even apoptosis (Orrenius et al., 2003) of the neurons. Moreover, during the synaptic long-term potentiation (LTP) learning process (Collingridge et al., 2004), newly synthesized NMDARs containing GluN2A subunits are introduced into the postsynaptic membrane.

Electrophysiological studies in rat brain slices at He HP showed a significant increase in the synaptic NMDAR response followed by postsynaptic excitability changes (Mor and Grossman, 2006, 2007). Other recent studies in oocytes expressing different receptor subtypes revealed that NMDARs containing the GluN2A subunit are the only ones that show current increase at He HP (Mor et al., 2012), while combinations with other GluN2 subunits show little inhibition or no current change. The magnitude of the current increase depends on the GluN1 subunit variant that is present in the receptor (Bliznyuk et al., 2015, 2016). Hyper-activation of the NMDARs containing the GluN2A subunit, which is the most abundant subunit in the adult CNS, could be the specific cause for the aforementioned neuronal death processes. Other iGluR members play only a very small part in inducing CNS hyperexcitation at HP; AMPAR (Pearce et al., 1994) and kainate receptors were unaffected by pressure. The inhibitory ionotropic GABA receptors were also insensitive to HP (Roberts et al., 1996), whereas inhibitory glycine receptors, while showing no effect on the maximal response, demonstrated a considerable increase of their IC_50_ at HP (Shelton et al., 1993). Given that HPNS is part of a general cellular HP stress response which entails simultaneous adaptive slowed CNS responses and opposing high frequency disturbances (Talpalar and Grossman, 2006) we hypothesized that NMDAR current augmentation at HP may result from inserting additional receptors (containing GluN2A subunit) into the membrane during the 20–25 min compression required for HP response stabilization (similar to the aforementioned LTP mechanism). To test our hypothesis, we used immunoprecipitation to detect any increase in NMDAR expression in the oocyte membrane, which is our most useful cellular model for direct testing of the NMDAR currents.

Following standard procedures, we were supposed to normalize NMDAR protein levels to those of “housekeeping” proteins such as glyceraldehyde-3-phosphate dehydrogenase (GAPDH) and/or β-actin. However, recent studies have revealed that GAPDH is a multifunctional protein rather than simply a glycolytic enzyme (for a review, see Hara et al., 2006; Tristan et al., 2011). Among those functions are modulation of the Ca^2+^ current component of AMPAR and IP3 receptors, regulation of microtubule bundling and cytoskeletal dynamics, membrane fusion, various functions of nuclear DNA, and even mediation of cell death. β-actin is also involved in various cytosolic functions such as mechanical support to maintain cell shape and movement, controlling cell-to-cell junctions, coordinating the movements of organelles such as mitochondria and Golgi vesicles, intracellular transport, and cell polarity (Alberts et al., 2002). Therefore, our second, no less important, aim was to test their cellular response to HP stress.

## Materials And Methods

### Oocyte Preparation

Stages V and VI oocytes were surgically removed from *Xenopus laevis* ovaries (anesthetized with 1.5 g/L ethyl 3-aminobenzoate methanesulfonate salt; Sigma-Aldrich, Israel), prepared and maintained in ND-96 solution (at 18°C) containing 96 NaCl, 2 KCl, 1 MgCl2, 1.8 CaCl2, 2.5 sodium pyruvate, and 5 HEPES (AMRESCO, LLC) mM; 10 mg/ml PEN/STREP, and 50 μg/ml gentamicin, adjusted to pH 7.5. The incisions were closed using absorbable sutures and the animals were returned to the tank. Surgery was performed according to the guidelines provided by the Ben-Gurion University of the Negev ethics committee for the care and use of animals for experimental work (IL-69-12-2011). Within 24 h of surgery, oocytes were injected with one of the two newly synthesized GluN1-1a splice variant cRNAs (5 ng) and the GluN2A subunit cRNA (5 ng), using a nanoliter injector (World Precision Instruments, Sarasota, FL, United States). All cRNAs were produced by Prof. M. Hollmann’s laboratory (Ruhr University, Bochum, Germany). The NMDAR cDNA accession numbers for GluN1-1a and GluN2A are U08261 and AF001423, respectively. Those and other NMDAR subtypes were successfully expressed in the oocytes’ membranes.

### Biotinylation

Three days after injection, the oocytes were labeled with Biotinylated Concanavalin A (Sigma C-2272) under normal (0.1 MPa) and He HP (5.0 MPa) conditions. HP conditions were attained by placing the oocytes in a bath inside the compression chamber. Under normal and pressure conditions oocytes were continuously perfused with fresh physiological solution containing 90 NaCl, 1 KCl, 1.5 BaCl_2_, 10 HEPES mM, and 10 μM biotinylated ConA, equilibrated with air, which was introduced by a high-pressure pump. In the present study [Ca^2+^]_o_ was substituted for by [Ba^2+^]_o_ in the physiological solution in order to avoid Ca^2+^-induced transient chloride currents, channel subconductances, and divalent cation-induced current inactivation (see Mor et al., 2012). Zero added [Mg^2+^] was used in order to remove its known voltage-dependent physiological blockade of the NMDARs to enable the receptor activation by perfused glutamate. Both changes were performed in the previous electrophysiological experiments (Bliznyuk et al., 2015, 2016).

### Pressure, Compression, and Decompression

The pressure chamber, perfusion system, and the experimental setup have been described in detail in Mor and Grossman (2006). Briefly, experiments were carried out in a pressure chamber (Canty Assoc., NY, United States). HP was attained by compressing helium for the experimental pressure range of 0.1–5.0 MPa. Rates of compression/decompression ranged between 0.1 and 1.0 MPa/min. In order to avoid transient temperature changes and mechanical shifts during compression (Grossman and Kendig, 1984), we used slow compression rate (0.1 MPa) at the beginning. When oocytes stabilized at about 1.0 MPa we gradually increased the compression rate concomitantly with the pressure increase up to 5.0 MPa. Experiments were conducted at a strictly controlled ambient temperature (25 ± 1°C). Typically, 20–25 min was needed to reach 5.0 MPa, achieve stable temperature conditions, and stabilize temperature transients of ±1–3°C during compression and decompression. We routinely follow this pressure protocol in all our experiments to demonstrate HP effects.

### Western Blotting

**FIGURE 1:**
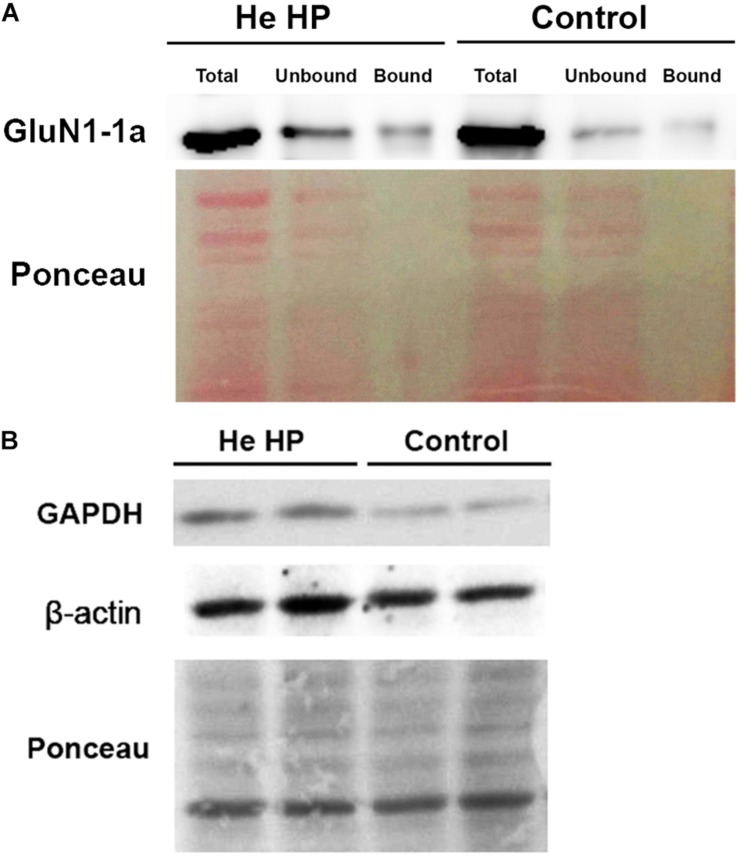
Immunoblot assay example and Ponceau staining of Western blot. **(A)** Immunostained GluN1-1a bands (above) and the Ponceau-stained full blot (below) showing total, bound, and unbound fractions under control and He HP conditions. **(B)** Immunostained GAPDH and β-actin bands of the total fraction (above) and the Ponceau-stained full blots (below) under control and He HP conditions.

Oocytes were homogenized in H-buffer containing 100 mM NaCl, 20 mM Tris–HCl pH 7.4, 1% Triton X-100, and protease inhibitor (Sigma P-2714) cocktail, 60 ± 5 min after the beginning of the compression. Streptavidin-agarose beads (Sigma S-1638) were used to precipitate biotinylated proteins from homogenates of single oocytes, which were then resolved with SDS-PAGE. To determine the expression of the GluN1-1a subunit, we used a working dilution of 1:4000 of rabbit polyclonal antibody (Thermo Fisher Scientific, Inc., United States, PA5-34599). To determine GAPDH expression we used working dilution of 1:5000 of rabbit polyclonal antibody (Santa Cruz Biotechnology, Inc., United States, GAPDH sc-25778) and β-actin expression we used working dilution of 1:10 000 of mouse polyclonal antibody (Sigma-Aldrich, Israel). Detection of immunoreactive bands was carried out using enhanced chemiluminescence (Biological Industries, Israel). The intensity of the bands (including Ponceau staining, see Figure 1) was measured using ImageJ software (NIH, Bethesda, MD, United States) (Schneider et al., 2012). The original pictures of the blots can be found in the Supplementary Figures S1–S4.

All protein blots were normalized to Ponceau staining for each individual lane. Each individual lane was stained for all Ponceau S-bands and the total intensity of all bands was taken as 100% (total protein intensity). The immunostained protein band intensity was divided by total protein intensity. Data are presented as mean intensity ± SEM; n denotes the number of successful experiments (number of oocytes) for each experimental protocol. The degree of significance was denoted by the values of *p* (two-tail Student’s *t*-test for two populations) using Microsoft Excel software. Results were considered statistically different when *p* < 0.05.

## Results

### GAPDH and β-Actin Protein Levels Increased at He HP

**FIGURE 2:**
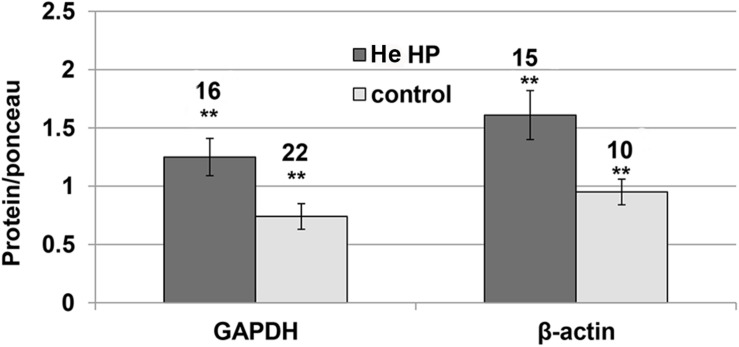
Quantitative analysis of GAPDH and β-actin protein levels at He HP. Protein levels were normalized to Ponceau staining (calculated for each oocyte and averaged); data indicate significant concomitant increase of GAPDH and β-actin at He HP. The values indicate number of oocytes tested; error bars ± SEM. ^∗∗^ degree of statistical significance (*p* < 0.01) of *t*-test (0.1 MPa vs. 5.0 MPa).

It became clear in the study’s early stages that normalizing NMDAR protein levels to those of GAPDH and β-actin markers was impossible since their levels were pressure-dependent. Therefore, the results of both markers and the NMDAR proteins were normalized to total Ponceau protein staining. We observed a significant GAPDH increase (blot example Figures 1B, 2) from a control value of 0.74 ± 0.11 (*n* = 22) to He HP value of 1.25 ± 01.6 (*n* = 16; *p* = 0.01). Concomitantly, β-actin also increased (blot example Figures 1B, 2) from a control value of 1.06 ± 0.18 (*n* = 8) to He HP value of 1.81 ± 0.19 (*n* = 7; *p* = 0.01).

### The Protein Level of the GluN1-1a + GluN2A Subtype Is Not Affected by He HP

**FIGURE 3:**
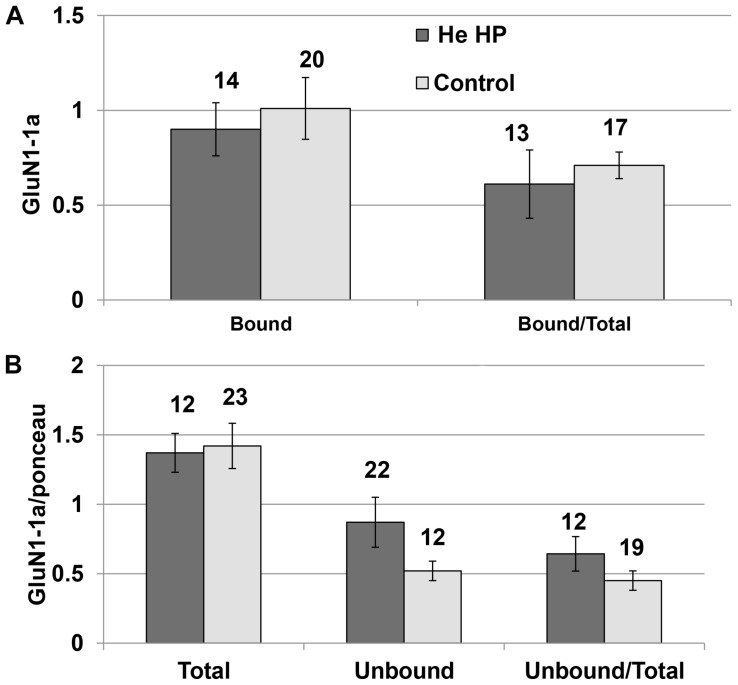
Quantitative analysis of NMDAR protein levels at He HP. **(A)** Protein levels were calculated for each oocyte and averaged. Data indicate no significant increase in the bound fraction (receptors present in the oocyte membrane) at He HP. The values indicate number of oocytes tested; error bars ± SEM. **(B)** Protein levels were normalized to the total Ponceau-stained protein (calculated for each oocyte and averaged). Data indicate no significant increase in total or unbound fractions at He HP. The values indicate number of oocytes tested; error bars ± SEM.

Immunoprecipitation of the NMDARs (GluN1-1a + GluN2A subtype) were conducted under control and pressure conditions. The results of the Western blotting (blot example Figures 1A, 3) indicated that there was no significant increase in total GluN1-1a protein (control: 1.37 ± 0.15, *n* = 16; HP: 1.24 ± 0.14, *n* = 12) or its membrane expression (control: 0.98 ± 0.12, *n* = 16; HP: 0.94 ± 0.17, *n* = 12) at He HP.

## Discussion

The consistent increase in GAPDH and β-actin proteins under hyperbaric conditions strongly suggests the presence of a general cellular stress response to HP. Morris et al. (2015) showed a significant increase in differential gene expression in response to acute pressure and temperature exposures in shallow-water shrimp. HP significantly augmented the transcription of gene coding for NMDARs, ADP ribosylation factor, two heat shock proteins, β-actin, and GAPDH. The latter findings in the crustacean nervous system strongly suggest that a similar increase in the relevant mRNAs was indeed translated to protein levels in frog oocytes exposed to similar HP conditions. In contrast, the immunoprecipitation experiments did not indicate any increase in surface expression of NMDARs in the oocyte membrane under HP conditions. Similarly, there was no significant change in total or unbound protein levels, suggesting that there was no increase in NMDAR synthesis or storage. In these experiments pressure always induced current increase disregard of the cRNA quantity. However, one should bear in mind that our experiments were carried out with oocytes over-expressing the NMDAR, after they had been injected with the required cRNAs (see section “Materials and Methods”). Considering the increased transcription of NMDAR in crustaceans (unspecified, Morris et al., 2015), it is possible that under true physiological conditions, some increase of the innate NMDAR production/storage/membrane insertion occurs. Previous experiments under similar conditions (Bliznyuk et al., 2015, 2016) eliminated aggregate formation, modified stoichiometry, and glutamate/glycine affinity changes of NMDARs as causes for the current increase of NMDARs containing the GluN2A subunit. Those results taken together with the present ones may suggest that the NMDAR current augmentation is mediated by molecular changes in a single receptor response to HP. These changes could be mediated directly by He HP effect on the 3D protein microstructure of the receptor that may affect its pore conductance, as we showed in our late molecular dynamic simulation study (Bliznyuk et al., 2019). Alternatively, similar changes induced indirectly by pressure effects on the interaction of the phospholipid bilayer membrane with the embedded protein. Additional indirect mechanism(s) could be HP modulation of NMDAR Mg^2+^ and Zn^2+^ blockade or Ca^2+^ permeability regulated by phosphorylation (Dingledine et al., 1999; Skeberdis et al., 2006; Traynelis et al., 2010; Paoletti et al., 2013). The GAPDH increase at HP may suggest other NMDAR control mechanisms are involved. GAPDH interacts with the synthesizing enzyme of D-serine, elevating its concentration (Suzuki et al., 2015). D-serine, which, like glycine, is a co-agonist of NMDAR activation, could increase the receptor’s Ca^2+^ current. Furthermore, after GAPDH loses its glycolytic activity, it can interact with GluA2 – a subunit of AMPAR – and dramatically increase its Ca^2+^ conductance, which is normally close to zero (Wang et al., 2012). Thus, together with the potentiated response of NMDAR, they could mediate serious glutamate excitotoxicity. Support for this model is provided by the early findings that AMPAR blockers may at least partially protect against high pressure-induced hyperexcitability (Pearce et al., 1994). The observed β-actin increase, on the other hand, could be a preventive measure to anticipated mechanical stress caused by increased pressure, which at much higher values of greater than 29 MPa, severely damages (depolymerizes) various types of actins (Bourns et al., 1988; Daniels and Grossman, 2010).

We assume that HPNS arises from dysfunction of network synaptic activity. The NMDAR has an important role in neuronal excitatory transmission within the CNS. This study combined with previous research (Mor et al., 2012; Bliznyuk et al., 2015, 2016) shows that the various NMDAR subunits have differential responses to HP. The most abundant and important subunit in the CNS excitatory synaptic transmission, GluN2A, is the only one that shows an increased current in response to HP when it is co-expressed with most of the GluN1 subunit variants. The augmented NMDAR currents increase glutamatergic synaptic activity and cause neuronal dendritic hyperexcitability, which constitutes one of HPNS’s main features. HPNS symptoms are usually reversible upon decompression. However, recent reports (Gronning and Aarli, 2011) have intriguingly suggested that repetitive exposure over years to HP may cause chronic memory and motor impairment in professional divers. Possibly having been exposed to HP either just below the threshold or even above the HPNS normal threshold, these divers’ HPNS symptoms were antagonized by the use of narcotic gas mixtures (such as Trimix containing O_2_, N_2_, and He). We hypothesize that even if clear HPNS symptoms were not observed, the glutamate NMDAR response was still potentiated, causing more Ca^2+^ ion flow into the neurons (possibly with the contribution of the aforementioned GluA2-containing receptor). An overload of Ca^2+^ can activate metabolic cascades through several signal transduction pathways, deteriorating the neuron and possibly eventually leading to cell death via apoptosis (Orrenius et al., 2003). The long-term health effects are part of HPNS, suggesting an accumulation of minute excitotoxic damage events inflicted by the potentiation of NMDARs and conversion of AMPARs to Ca^2+^-conducting receptor variants during each deep dive. The constellation of symptoms and signs of HPNS supports the idea that the permanent damage could spread to various parts of the brain. Finally, GAPDH increase resulting from HP and other physiological stress responses might be associated with translocation of various factors to the nucleus, allowing for modified control of DNA transcription, DNA replication, and DNA repair (see a review, Hara et al., 2006).

Understanding the precise mechanism(s) of NMDAR hyperexcitation, GAPDH and β-actin contribution to nuclear and cytosolic toxic processes during HP exposure could provide the key for eliminating the adverse, yet reversible, short-term effects of HPNS and, hopefully, the deleterious long-term consequences.

## Data Availability Statement

All datasets generated for this study are included in the manuscript/Supplementary Files.

## Ethics Statement

The animal study was reviewed and approved by Ben-Gurion University of the Negev.

## Author Contributions

AB designed the research study, performed the research, analyzed the data and wrote the manuscript. AB and YG interpreted the data. YG and MH conducted a critical revision of the manuscript.

## Conflict of Interest

The authors declare that the research was conducted in the absence of any commercial or financial relationships that could be construed as a potential conflict of interest.

## Abbreviations

AMPAR: α-amino -3-hydroxy-5-methyl-4-isoxazolepropionic acid receptor
ANS: autonomic nervous system
CNS: central nervous system
GAPDH: glyceraldehyde-3-phosphate dehydrogenase
HP: high pressure
HPNS: high pressure neurological syndrome
iGluR: ionotropic glutamate receptor
LTP: long term potentiation
NMDR: *N*-methyl -D-aspartate receptor.
