# Supplementary material for: High Pressure Stress Response: Involvement of NMDA Receptor Subtypes and Molecular Markers
Source: Front Physiol. 2019 Sep 27;10:1234. doi: 10.3389/fphys.2019.01234 (PMC6777016; doi:10.3389/fphys.2019.01234)
Supplement: Supplementary file 1 [file Image_1.PDF]

Supplementary Material

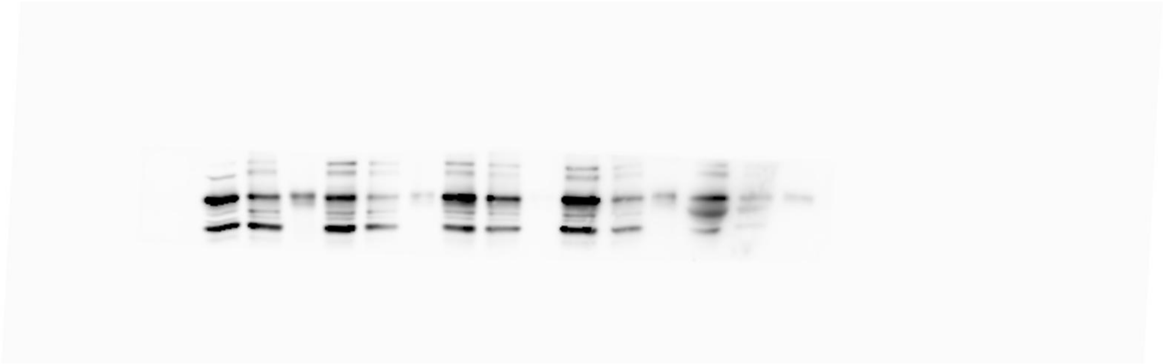

Supplementary Figure 1. The original picture of the GluN1-1a blot from figure 1A.

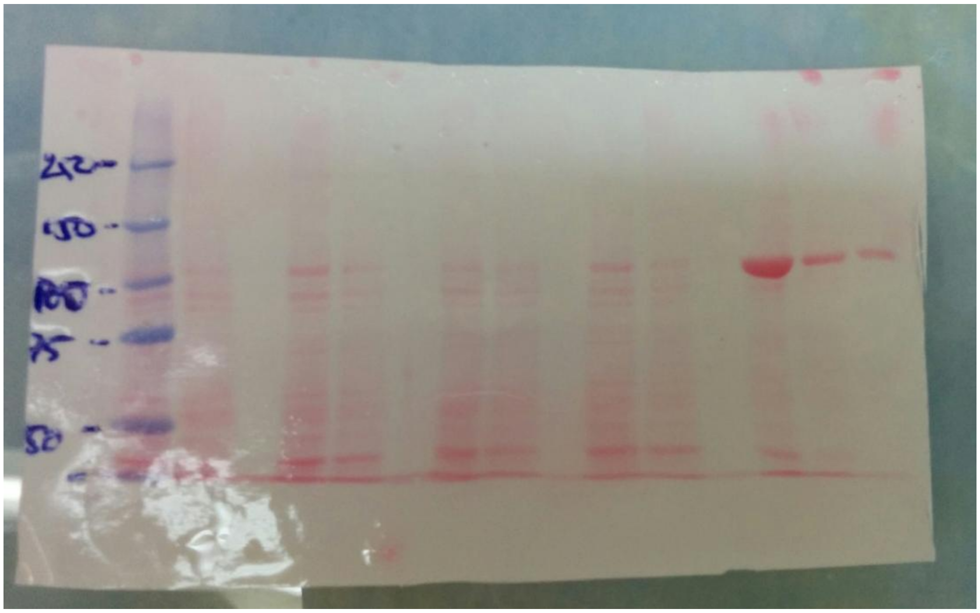

Supplementary Figure 2. The original picture of the ponceau staining from figure 1A.

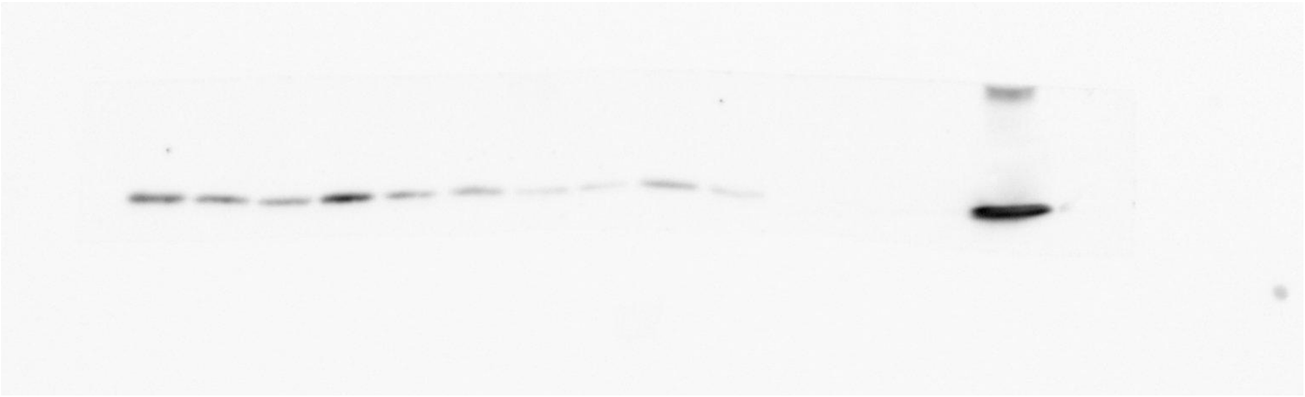

Supplementary Figure 3. The original picture of the GAPDH blot from figure 1B.

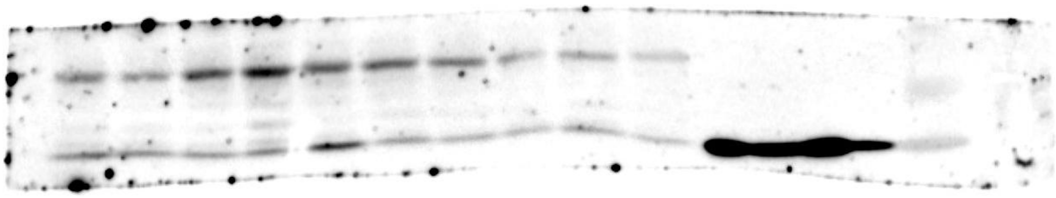

Supplementary Figure 4. The original picture of the β-actin blot from figure 1B.

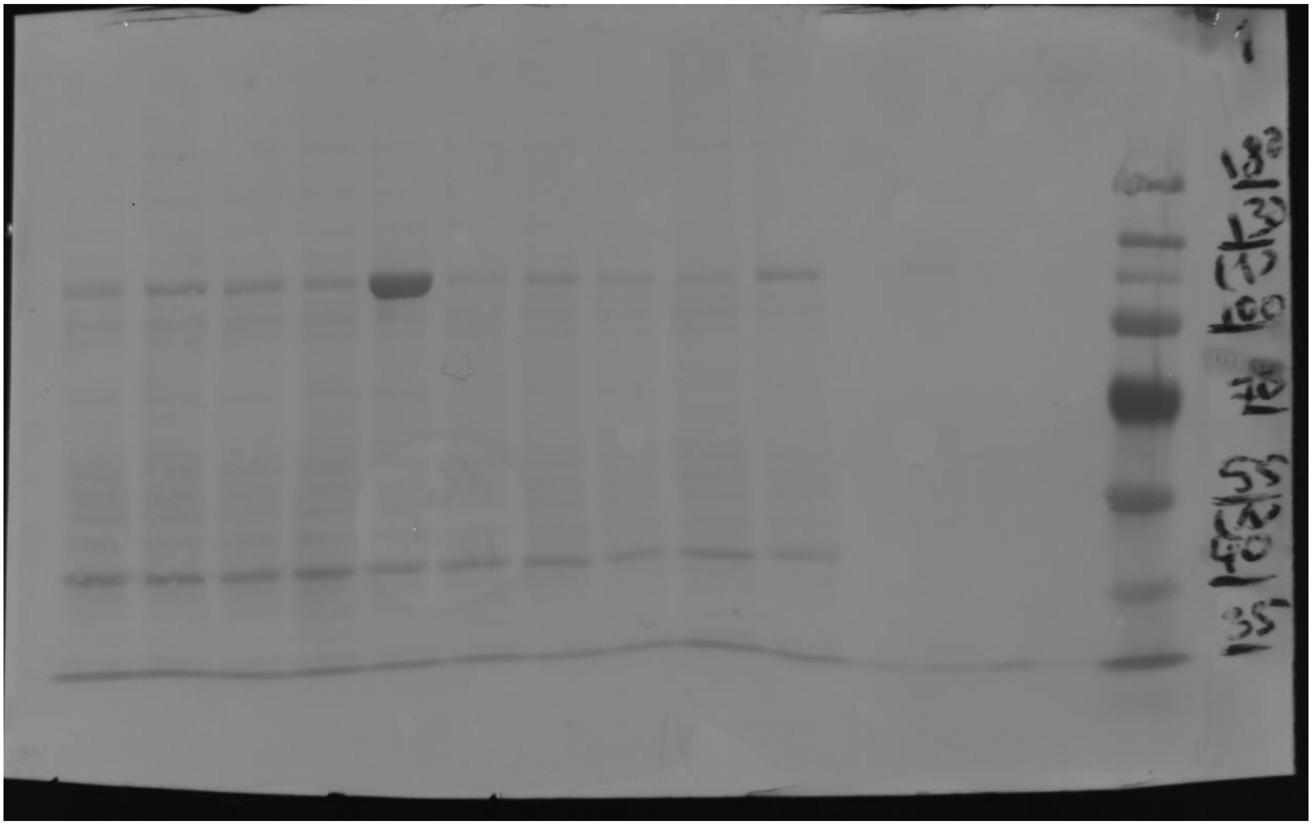

Supplementary Figure 2. The original picture of the ponceau staining from figure 1B.
